# Assessment of Phoenix Sepsis Score, pSOFA, PELOD-2, and PRISM III in Pediatric Intensive Care

**DOI:** 10.3390/children12030262

**Published:** 2025-02-21

**Authors:** Adriana Hadzhieva-Hristova, Darina Krumova, Temenuga Stoeva, Ralitza Georgieva, Violeta Iotova

**Affiliations:** 1 Department of Pediatrics, Medical University of Varna, 9002 Varna, Bulgaria; dr.krumova@abv.bg (D.K.); violeta.iotova@mu-varna.bg (V.I.); 2 First Clinic and PICU, St. Marina University Hospital, 9010 Varna, Bulgaria; 3 Department of Microbiology and Virology, Medical University of Varna, 9002 Varna, Bulgaria; temenuga.stoeva@abv.bg; 4 Microbiology Laboratory, St. Marina University Hospital, 9010 Varna, Bulgaria; 5 Department of Neonatology, Medical University of Sofia, 1431 Sofia, Bulgaria; rgeorgieva@medfac.mu-sofia.bg

**Keywords:** prediction scores, Phoenix Sepsis Score, PRISM III, PELOD-2, pSOFA, discrimination, calibration, children, complications, PICU

## Abstract

**Background/Objectives**: Early identification of pediatric sepsis complications in intensive care is challenging and requires improved diagnostic tools. This study aimed to compare the Phoenix Sepsis Score (PSS), pSOFA, PELOD-2, and PRISM III in assessing clinical complexity in children with septic and critical conditions in the PICU and to identify the most suitable scale for this patient cohort. **Methods**: Data were collected prospectively from 53 children between June 2022 and January 2024. Patients were categorized into septic (n = 42) and non-infectious SIRS (n = 11) and further classified by outcome—with/without complications (n = 23/30). The predictive accuracy of the scoring systems was evaluated by discrimination and calibration and by recalibration for the PSS for improved performance. **Results**: Respiratory (18.8%) and neurological complications (9.4%) were the most common adverse events. Clinical deterioration was observed in 43.4% of cases, including one fatality. Patients with complications stayed longer in the PICU (14 ± 10 days). In the patients with complications, all scoring systems had higher median values. Only PSS showed a significant difference (*p* = 0.0023). PSS demonstrated the highest overall predictive accuracy (76.2%) outperforming PRISM III (62.3%) and PELOD-2 (58.5%). The pSOFA scale showed high accuracy (88.0%) in identifying patients without complications. The strongest association was between chronic disease (hazard ratio Exp(B) = 1.718) and deteriorations, while mechanical ventilation suggested a reduced risk of complications (Exp(B) = 0.509). **Conclusions**: PSS showed superior predictive accuracy (76.2%) for deteriorations in pediatric patients with suspected infection and proved adaptable for further validation in larger populations.

## 1. Introduction

Advancements in medicine have significantly broadened the scope of pediatric intensive care, leading to improved survival rates among children who, in the past, would have had minimal or no chances of survival. Nearly six decades after the establishment of the first pediatric intensive care unit (PICU) in Switzerland [1], pediatric intensive care medicine has undergone significant improvements, which has contributed to a reduction in child mortality worldwide [2]. Early identification of initial symptoms in children, who exhibit signs of clinical decompensation, is crucial for optimizing the diagnostic process, minimizing long-term complications, and improving patient outcomes [3]. Critically ill children demonstrate significant deviations from normal physiological homeostasis, characterized by disruptions in fundamental vital functions, including respiratory and cardiovascular activity, renal performance, and endocrine regulation [4]. These alterations can be quantified using various scoring systems designed to assess the severity of the condition, which are developed based on deviations of physiological parameters from normative values [5,6]. The scoring systems serve as standardized tools to quantify disease severity, evaluate the risk of morbidity or mortality, and monitor the clinical status of patients. By integrating a range of physiological, laboratory, and clinical parameters into a composite score, they attempt to provide a comprehensive reflection of the patient’s current health status [7]. Disease severity scoring systems must be easy to use, cost-effective, and universally applicable across different healthcare departments [8].

The Pediatric Risk of Mortality Scale (PRISM III) is one of the most widely used tools in pediatric intensive care for objectively assessing disease severity in critically ill children. Its multidimensional approach supports the diagnosis, clinical monitoring, and outcome prediction in critical care [9].

The Pediatric Logistic Organ Dysfunction-2 (PELOD-2) scale is designed to assess the severity of organ dysfunction in critically ill children who are admitted to intensive care units. It also serves as a valuable tool for monitoring disease progression and evaluating patient outcomes during intensive care [10].

In 2017, the Pediatric Sequential Organ Failure Assessment (pSOFA) scale was validated, primarily developed for use in patients with suspected or confirmed infections, within the framework of the Sepsis-3 definition of sepsis [11].

The Phoenix Sepsis Score (PSS) is an innovative scale for the diagnosis and evaluation of pediatric sepsis, validated in January 2024. The definition is suspected or confirmed infection, accompanied by life-threatening organ dysfunction involving disturbances in four systems: respiratory, cardiovascular, nervous, and coagulation. Sepsis in children is diagnosed with a PSS score of ≥2, including at least 1 point for cardiovascular dysfunction, which classifies the condition as a septic shock [12].

The scores for assessing a patient’s status are broadly classified into two main categories: *prognostic* and *descriptive*. Prognostic scales like PRISM III predict outcomes such as mortality, while descriptive scales like pSOFA and PELOD-2 evaluate organ dysfunction and identify patients at risk of deterioration [13]. In addition, the PRISM III and PELOD-2 scores can be converted into a probability of lethality, providing quantitative prognostic information. In contrast, the pSOFA scale does not offer the capability to predict mortality [8]. It is important to note that both pSOFA and PSS are specifically validated for use in patients with suspected or confirmed infections. Their predictive value in other clinical conditions may vary and could potentially be less accurate [14,15].

Current assessment systems often overlook dynamic changes in pediatric vital signs, leading to potential misdiagnoses and delayed care. While useful as decision-support tools, they cannot replace clinical judgment. Integrating indicators such as biomarkers could improve the prognostic accuracy and could enable earlier detection of patient deterioration [16].

The aim of this study was to evaluate the effectiveness of PRISM III, PELOD-2, pSOFA, and PSS in assessing clinical complexity in children with septic and critical conditions in a pediatric intensive care unit and to determine the most suitable scale for use in this cohort.

## 2. Materials and Methods

### 2.1. Study Methodology, Patients, and Clinical Outcomes

Between 1 June 2022 and 31 January 2024, a prospective study was conducted in the pediatric intensive care unit of a University Hospital in Bulgaria, involving 53 children with an acute onset of infectious diseases and/or critical conditions. Patients, aged 7 days to 18 years, were included based on predefined criteria and categorized into two groups:

- *Septic group:* children with systemic inflammatory response syndrome (SIRS), caused by confirmed or suspected infection, meeting ≥2 criteria, one of which must include changes in body temperature or leukocyte count;
- *Critical group*: children with non-infectious SIRS.

Exclusion criteria encompassed patients with trauma, oncohematologic diseases, other forms of immune incompetence, surgical conditions, neonates in the early neonatal period (<7 days), patients with a hospital stay exceeding 15 days, and pregnancy.

The definition of pediatric sepsis validated in the International Pediatric Sepsis Consensus of 2005 was applied when categorizing the septic group [17].

Detailed clinical data were collected using a structured questionnaire, which was designed based on a systematic review and tailored to the objectives and hypotheses of the study (Supplementary File S1). The questionnaire was conducted as a structured interview with the patient and their parent after obtaining informed consent (IC) from the parent, guardian, or custodian.

The physical examination was performed using standard propaedeutic methods. The patient’s general status was assessed within 24 h of PICU admission using PRISM III, pSOFA (via Pediatric Scores Plus app version 7), PELOD-2, and the Phoenix Sepsis Score, with scores being calculated using publicly available domains. PSS was retrospectively derived, and both PSS and pSOFA were assessed only in septic patients [18,19].

The PRISM III scale is calculated based on the following variables: systolic blood pressure, heart rate, body temperature, mental status, pupillary responses, acidosis, pH, pCO2, total CO2, PaO2, glucose, potassium, serum creatinine, urea, leukocyte count, platelet count, prothrombin time (PT), and partial thromboplastin time (PTT). The maximum achievable score on the scale is 74.

The PELOD-2 score is calculated based on the values of the following variables: mean arterial pressure, lactate levels, Pediatric Glasgow Coma Scale (pGCS), pupillary response, PaO_2_/FiO_2_ ratio, PaCO_2_, need for invasive ventilation, white blood cell count, platelet count, and serum creatinine. The possible score ranges from 0 to 33 points.

The pSOFA scale incorporates indicators to assess organ dysfunction in children, including serum bilirubin, platelet count, pGCS, and age-adjusted values for serum creatinine and mean airway pressure. The scale allows for the calculation of a total score, with a maximum value of 24 points.

The Phoenix Sepsis Score can be calculated even when certain parameters are unavailable. For instance, if lactate levels are not measured or vasoactive medications are not used, the cardiovascular component of the score can still be determined using blood pressure values. Parameters that are not assessed or applied do not contribute to the final score [15].

Patients were monitored until discharge and classified into two groups: those with complications and those without complications, based on the presence or absence of documented clinical deterioration during the course of the disease.

### 2.2. Laboratory Tests

Hospitalized children underwent venipuncture for the collection of complete blood count (CBC), blood gas analysis, coagulation, and biochemical parameters, including C-reactive protein (CRP), procalcitonin (PCT), blood glucose, and serum electrolytes. Blood cultures were obtained prior to the initiation of antibiotic therapy. Laboratory parameters were analyzed using automated systems, including the Sysmex XN 1000 (Sysmex Corporation, Kobe, Japan), Sysmex CS-2500 (Sysmex Corporation, Kobe, Japan), SIEMENS ADVIA 1800 (Siemens Healthineers, Erlangen, Germany), and GEM Premier 3000 (Instrumentation Laboratory, Bedford, MA, USA). The BACTEC 9050 automated system (BD, Franklin Lakes, NJ, USA) was used for microbial isolation from blood samples, with incubation for 5 days, extended up to 14 days in cases of suspected fungal infection. The positive blood cultures were subjected to rapid identification by a MALDI-TOF-MS Sepsityper kit (Bruker, Daltonics, Billerica, MA, USA) and MALDI-TOF-MS instrument (Bruker, Daltonics).

### 2.3. Statistical Analysis

Statistical analyses were performed using IBM Statistical Package for the Social Sciences, version 24 (SPSS Inc., Chicago, IL, USA); GraphPad Prism, version 10.4.0 (GraphPad Software, San Diego, CA, USA); and Python, version 3.13.0 (Python Software Foundation, Beaverton, OR, USA) for graphical representation. Descriptive statistics for parametric quantitative data included calculations of mean, standard deviation (SD), and minimum and maximum values. Non-parametric quantitative data were summarized using medians and interquartile ranges (IQRs). Qualitative data were presented as frequencies and percentages.

Normality of data distribution was assessed using the Kolmogorov–Smirnov and Shapiro–Wilk tests. For the analysis of non-normally distributed and non-parametric data, the Mann–Whitney U test was applied. Statistical significance was set at *p* ≤ 0.05.

Our study focused on evaluating the discriminative ability and calibration of the scoring systems as two essential metrics for assessing their performance. Discriminative ability refers to the capacity of a scale to distinguish between patients with different clinical outcomes. Calibration, on the other hand, assesses the accuracy of predicted probabilities compared with the observed outcomes.

When a model, such as the Phoenix Sepsis Score, is validated on a specific cohort such as children with suspected infection, there is a potential for underestimating or overestimating the risk in different populations. Recalibration addresses these discrepancies by adjusting the model, thereby reducing systematic bias and improving predictive accuracy. These evaluations are crucial for determining the reliability and performance of scoring systems in predicting patient outcomes [20,21].

The performance of each model was evaluated using the area under the receiver operating characteristic (ROC) curve to predict specific outcomes, such as the development of complications. The analysis of ROC curves included the calculation of the area under the curve (AUC), sensitivity, specificity, positive predictive value (PPV), and negative predictive value (NPV). According to the established benchmarks, an AUC value of 0.7 indicates acceptable discrimination, 0.8 signifies good discrimination, and 0.9 represents excellent discrimination [20].

The calibration of the predicted probabilities was assessed by comparing observed and predicted event probabilities. This process involved calculating predicted values from the model, stratifying them into decile categories, and analyzing the observed frequencies within each category. To quantify calibration, the Hosmer–Lemeshow test was used as a statistical goodness-of-fit test, comparing predicted and observed probabilities. A result of *p* ≥ 0.05 was considered indicative of acceptable calibration, while significant deviations from this threshold suggested potential issues with the model calibration [20]. To improve the performance of models that did not calibrate well to our population, recalibration was performed.

The Cox proportional hazards model was applied to analyze the association between independent variables and the risk of events in the studied patient population. Statistical significance was defined as *p* < 0.05 based on results from a Wald test. The hazard ratio, represented as Exp(B), interprets relative risk: a value >1 indicates an increased risk, <1 indicates a decreased risk, and ≈1 suggests no significant effect [22].

This study was approved by the Ethics Committee of the Medical University of Varna (Protocol No. 115/31 March 2022).

## 3. Results

Over an 18-month period, 53 children meeting the pre-specified inclusion criteria were studied. The mean age of the hospitalized patients was 44 ± 56 months. The septic group comprised 42 patients, while the group with SIRS of non-infectious origin included 11 patients.

The data analysis revealed that 41.5% (22/53) of cases were diagnosed with lower respiratory tract infections with microbiologically confirmed causative agents including *Streptococcus pneumoniae*, *Mycobacterium tuberculosis*, and SARS-CoV-2. Gastrointestinal infections were the second most common with a prevalence of 11.3% (6/53). Identified pathogens included *Rotavirus*, *Salmonella Group D*, and *Clostridioides difficile*. A laboratory-confirmed bloodstream infection caused by *Escherichia coli* with a primary urinary tract origin was identified in one septic patient.

Among children with non-infectious SIRS, the highest relative proportion was attributed to intoxications (n = 4) and endocrine or metabolic disorders, such as diabetes mellitus with initial diabetic ketoacidosis (n = 2). The median length of stay in our study was 11 ± 8 days. Mechanical ventilation was required in 15% (n = 8) of hospitalized patients, and underlying chronic conditions were recorded in 24.5% (n = 13) of cases.

A total of 23 cases of clinical deterioration (43.4%) were reported across the studied groups, including one fatality (1/53). The distribution of secondary pathological conditions is summarized in Table 1.

Respiratory and neurological complications were among the most common in the studied cohort, with prevalences of 18.8% and 9.4%, respectively. Pleural effusion was the most frequent respiratory diagnosis, observed in three septic patients (5.6%). Neurological diagnoses were identified in 3.8% (n = 2) of patients in the non-infectious SIRS group.

The clinical characteristics and outcomes of the patients with and without complications are summarized in Table 2.

Patients with complications had significantly longer stays at the PICU. Among the clinical scoring systems under evaluation, only the PSS demonstrated a statistically significant difference between the two groups (*p* = 0.0023), with higher median values being reported for all scores in patients with complications.

The prognostic accuracy of the scales in predicting the risk of developing complications is summarized in Table 3.

PRISM III demonstrated a comparable accuracy (47.8%) in correctly predicting complications as compared with that of PELOD-2 (43.5%). The pSOFA scale exhibited high accuracy in identifying patients without complications (88.0%), while the PSS was the most accurate in predicting septic patients who developed complications during hospitalization (70.6%). Overall, PSS demonstrated the highest predictive accuracy (76.2%) and exhibited good performance across both groups studied.

The discrimination and calibration of the four prognostic scales are summarized in Table 4.

Among the analyzed models, the Phoenix Sepsis Score showed the highest sensitivity (80.0%), NPV (80.0%) and discriminative ability (AUC 0.736, CI: 0.576–0.897). The pSOFA scale exhibited the highest specificity (88.0%) and PPV (66.7%). PRISM III showed moderate stratification ability (AUC 0.650), while PELOD-2 displayed lower sensitivity (43.5%) and discriminative performance (AUC 0.591) across the studied groups.

The calibration of the PSS, assessed with the Hosmer–Lemeshow test (χ^2^ = 5.752, *p* = 0.016), indicated the need for model improvement by recalibration (χ^2^ = 0.000, *p* = 1.000). These findings are also illustrated in Supplementary File S2.

The analysis, using the Cox proportional hazards model, revealed no statistically significant effect of any of the variables from time to event (Table 5).

The strongest observed effect, though not statistically significant, was linked to underlying chronic disease (hazard ratio Exp(B) = 1.718), while mechanical ventilation showed a potential risk reduction (Exp(B) = 0.509), but this was also not statistically significant.

## 4. Discussion

The reduction in children mortality over the past two decades represents one of the most significant achievements in global health. However, current data and research predominantly focus on mortality rates, while the long-term complications for affected children remain insufficiently studied [23]. Currently, no universally accepted protocol exists for the initial assessment of children admitted to intensive care units in Bulgaria. The adoption of a standardized protocol for the admission of critically ill pediatric patients could enable the early identification of life-threatening conditions, the optimization of therapeutic strategies, the reduction of long-term complications, and a decrease in child mortality.

Based on the analyzed data, PRISM III demonstrated a better predictive value compared with PELOD-2 in children with septic and critical conditions. However, both scales exhibited a similar number of false negative cases (12/23 and 13/23, respectively) in identifying patients with complications. This limitation may result in delayed interventions and an underestimation of the patient’s overall condition.

The pSOFA model missed a significant proportion of patients with complications, which reflects its low sensitivity for identifying high-risk cases with sepsis. In contrast, the PSS showed superior performance, successfully identifying a significantly higher proportion of patients with complications.

The results of the study demonstrated that PELOD-2 exhibited limited performance, characterized by low discriminative ability and sensitivity. These findings contrast with those of other studies, where PELOD-2 reported significantly higher values for the area under the curve (AUC: 0.90) and sensitivity (76%) [24]. The fact that PELOD-2 was validated in a single multicenter study involving nine pediatric intensive care units in France and Belgium [10] suggests that its predictive value may be limited in populations outside these specific clinical settings. This highlights the necessity of further validation of the scale in diverse geographic regions and among varied patient populations.

PRISM III demonstrated a predictive value comparable with that reported by Jhamb et al. (AUC: 0.701) [25]. These findings suggest that PRISM III remains a reliable predictor across diverse populations, although its discriminatory ability is moderate. The high specificity of pSOFA (88.0%) proves significantly useful for identifying patients without complications; however, its low sensitivity (35.3%) indicates that the model fails to detect a substantial proportion of hospitalized patients with actual complications. Incorporating additional clinical variables, such as laboratory markers of inflammation (e.g., C-reactive protein, procalcitonin, presepsin) [26], could potentially reduce the number of false negative cases and enhance the reliability of the model in predicting complications in septic patients.

The poor calibration of the PSS predictive model necessitated its re-evaluation for use in its original form within our population. However, it has been demonstrated that when a model exhibits sufficient discriminatory power, recalibration can enhance its accuracy and performance in a specific population. Adaptation of the PSS by recalibration of the calibration intercept (λb) and calibration slope (βb) resulted in a good model fit and adequate prediction of the risk of developing complications in patients [27].

Although chronic disease did not reach statistical significance in the Cox analysis, the results demonstrated a trend toward an increased risk of developing complications, with an estimated 71.8% increase in risk (Exp(B) = 1.718) among hospitalized patients with underlying conditions. This observation is further supported by a recorded fatal outcome in a patient with a severe pulmonary infection of undetermined etiology, occurring on the background of congenital neuromuscular disease. These findings highlight the importance of chronic diseases as a potential risk factor for complications in critically ill pediatric patients.

Mechanical ventilation did not reach statistical significance in our study. However, Exp(B) = 0.509 indicates a trend toward risk reduction. The study revealed that the prevalence of mechanical ventilation in our population (15%) was significantly lower compared with rates reported in other studies, which documented values of 23.3% and 68.8%, respectively [28,29]. These discrepancies may be attributed to specific characteristics of the study population or differences in the criteria for initiating mechanical ventilation. The findings highlight the need for further analyses to better understand these variations and their potential impact on patient prognosis.

The length of hospital stay in the current study was significantly longer than the stays reported in Portugal and Egypt—3 and 4 days, respectively [24,30]. This discrepancy may reflect differences in the severity of clinical cases within the studied populations or the specific characteristics of the healthcare systems involved. For instance, Gonçalves et al. attributed the shorter length of stay in Portugal to the higher proportion of patients recovering after surgical interventions, which typically require only a brief stay in an intensive care unit. These findings highlight the importance of tailoring patient management strategies and taking into consideration the local healthcare context when outcomes across different health systems are compared.

### Study Limitations

The findings of this study should not be considered representative of the general population, as the study design was limited to the analysis of a specific target group.

## 5. Conclusions

The Phoenix Sepsis Score emerged as the most appropriate tool for assessing the risk of complications in patients with suspected infections. In comparison, pSOFA, PELOD-2, and PRISM III were less specific in identifying organ dysfunction in our pediatric population. While the sample size and single-center design may influence the universality of the results, the data provide valuable insights for future research. Future studies with larger, multicenter cohorts are needed to refine risk stratification tools, ultimately improving patient outcomes in both septic and non-septic critically ill children.

## Figures and Tables

**Table 1:** Distribution of complications among septic and critical patients.

Diagnosis ^§^	All n (%)	Septic n (%)	Non-Infectious SIRS n (%)
**Respiratory complications**	**10 (18.8)**	**10 (18.8)**	**0 (0.0)**
ARDS ^†^	1 (1.9)	1 (1.9)	0 (0.0)
Hydropneumothorax	1 (1.9)	1 (1.9)	0 (0.0)
Pleural effusion with lung abscess	2 (3.8)	2 (3.8)	0 (0.0)
Pleural effusion	3 (5.6)	3 (5.6)	0 (0.0)
Pyothorax	2 (3.8)	2 (3.8)	0 (0.0)
Tuberculous pleuritis	1 (1.9)	1 (1.9)	0 (0.0)
**Neurological complications**	**5 (9.4)**	**3 (5.6)**	**2 (3.8)**
Febrile seizure	1 (1.9)	1 (1.9)	0 (0.0)
Cerebral edema	1 (1.9)	1 (1.9)	0 (0.0)
Encephalitis	1 (1.9)	1 (1.9)	0 (0.0)
Cerebral infarction	1 (1.9)	0 (0.0)	1 (1.9)
Intracerebral hemorrhage *	1 (1.9)	0 (0.0)	1 (1.9)
**Other complications**	**8 (15.2)**	**4 (7.6)**	**4 (7.6)**
Urosepsis	2 (3.8)	2 (3.8)	0 (0.0)
Hemolytic uremic syndrome	1 (1.9)	1 (1.9)	0 (0.0)
Upper limb soft tissue abscess *	1 (1.9)	1 (1.9)	0 (0.0)
Anasarca **	1 (1.9)	0 (0.0)	1 (1.9)
Diabetic ketoacidosis	2 (3.8)	0 (0.0)	2 (3.8)
Esophageal stenosis	1 (1.9)	0 (0.0)	1 (1.9)
**Patients with organ dysfunction**	**23 (43.4)**	**17 (32.1)**	**6 (11.3)**
**Patients without organ dysfunction**	**30 (56.6)**	**25 (47.2)**	**5 (9.4)**
**Total**	**53 (100)**	**42 (79.3)**	**11 (20.7)**

^§^—Interpreted as complications during assessment; ARDS—acute respiratory distress syndrome; ^†^—complication resulting in a fatal outcome; * refers to a newborn; ** patient with nephrotic syndrome.

**Table 2:** Analysis of clinical characteristics and scores in patients with/without complications.

Variables	Complicated Patients	Not Complicated Patients	*p*
Mean age (months) ± SD	53 ± 51	36 ± 59	0.0652
Gender (male/female)	12/11	13/17	0.5862
Underlying diseases	4 (17.4)	9 (30.0)	0.3487
Fever	16 (69.6)	21 (70.0)	>0.9999
Mechanical ventilation	5 (21.7)	3 (10.0)	0.2720
Length of PICU stay (days) ± SD	14 (±10)	8 (±5)	**0.0001**
Total	23 (43.4)	30 (56.6)	**<0.0001**
**Scores**	**Median (IQR)**	**Median (IQR)**	***p***
PRISM III	6 (2–19)	2 (2–7.750)	0.1442
PELOD-2	3 (1–8)	2 (1–5)	0.3387
pSOFA *	5 (3–9)	4 (3–6)	0.2627
PSS *	2 (1–2)	1 (1–1)	**0.0023**

SD—Standard deviation; IQR—interquartile range; PRISM III—Pediatric Risk of Mortality III; PELOD-2—Pediatric Logistic Organ Dysfunction-2; pSOFA—Pediatric Sequential Organ Failure Assessment; PSS—Phoenix Sepsis Score; * calculated for the septic group; values in bold are statistically significant.

**Table 3:** Prognostic accuracy of PRISM III, PELOD-2, pSOFA, and Phoenix Sepsis Score.

	Observed	Patients	Total *	OPA %
Predicted by Score	Without OD n (%)	With OD n (%)		
**PRISM III**				
Without OD n (%)	22 (73.3) [TN]	8 (26.7) [FN]	30 (56.6)	63.3%
With OD n (%)	12 (52.2) [FP]	11 (47.8) [TP]	23 (43.4)	
Total *	34 (64.1)	19 (35.9)	53 (100)	
**PELOD-2**				
Without OD n (%)	21 (70.0) [TN]	9 (30.0) [FN]	30 (56.6)	58.5%
With OD n (%)	13 (56.5) [FP]	10 (43.5) [TP]	23 (43.4)	
Total *	34 (64.1)	19 (35.9)	53 (100)	
pSOFA **				
Without OD n (%)	22 (88.0) [TN]	3 (12.0) [FN]	25 (59.5)	66.7%
With OD n (%)	11 (64.7) [FP]	6 (35.3) [TP]	17 (40.5)	
Total *	33 (78.6)	9 (21.4)	42 (100)	
PSS **				
Without OD n (%)	20 (80.0) [TN]	5 (20.0) [FN]	25 (59.5)	76.2%
With OD n (%)	5 (29.4) [FP]	12 (70.6) [TP]	17 (40.5)	
Total *	25 (59.5)	17 (40.5)	42 (100)	

OD—Organ dysfunction; OPA—overall predictive accuracy; TN—true negative; FN—false negative; FP—false positive; TP—true positive; PSS—Phoenix Sepsis Score; * the percentage was calculated from the total number of patients (n = 53); ** calculated from the septic group.

**Table 4:** Assessment of discrimination and calibration of four prognostic scores.

Metrics	PRISM III	PELOD-2	pSOFA *	Phoenix Sepsis Score *
Sensitivity (%)	47.8	43.5	35.3	**80.0**
Specificity (%)	73.3	70.0	**88.0**	70.6
PPV (%)	57.9	52.6	66.7	70.6
NPV (%)	64.7	61.8	66.7	80.0
AUC (CI)	0.650 (0.499–0.801)	0.591 (0.432–0.749)	0.587 (0.401–0.773)	0.736 (0.576–0.897)
Hosmer–Lemeshow test, χ^2^	0.472 (*p* = 0.790)	2.308 (*p* = 0.511)	4.068 (*p* = 0.254)	5.752 (***p* = 0.016**)

PPV—Positive predictive value; NPV—negative predictive value; AUC—area under the curve; CI—confidence interval; * assessed only for the septic group Hosmer–Lemeshow test; χ^2^—chi-square, *p* > 0.05.

**Table 5:** Cox regression analysis.

Independent Variable	B	SE	Wald	df	*p*	Exp (B)
Age	0.005	0.005	0.997	1	0.318	1.005
Gender	−0.264	0.502	0.276	1	0.599	0.768
Underlying diseases	0.541	0.605	0.801	1	0.371	1.718
Mechanical ventilation	−0.676	0.601	1.263	1	0.261	0.509

B—Coefficient; SE—standard error; Wald—Wald test; df—degrees of freedom; *p*-value; Exp(B)—hazard ratio, *p* < 0.05.

## Data Availability

The data presented in this study are openly available in https://doi.org/10.7910/DVN/9IVA8B.

## Supplementary Materials

The following supporting information can be downloaded at: https://www.mdpi.com/article/10.3390/children12030262/s1, Supplementary File S1: Clinical Questionnaire; Supplementary File S2: Calibration and recalibration of the scores.

## Abbreviations

The following abbreviations are used in this manuscript:

PICU	Pediatric intensive care unit
PRISM III	Pediatric Risk of Mortality Scale
PELOD-2	The Pediatric Logistic Organ Dysfunction-2
pSOFA	Pediatric Sequential Organ Failure Assessment
PSS	The Phoenix Sepsis Score
SIRS	Systemic inflammatory response syndrome
IC	Informed consent
pH	Potential of hydrogen
pCO_2_	Partial pressure of carbon dioxide
PaO_2_	Partial pressure of oxygen
PT	Prothrombin time
PTT	Partial thromboplastin time
pGCS	Pediatric Glasgow Coma Scale
PaO_2_/FiO_2_ ratio	Partial pressure of arterial oxygen to fraction of inspired oxygen ratio
PaCO_2_	Partial pressure of carbon dioxide in arterial blood
SpO_2_/FiO_2_ ratio	Oxygen saturation to fraction of inspired oxygen ratio
INR	International normalized ratio
CBC	Complete blood count
CRP	C-reactive protein
PCT	Procalcitonin
MALDI-TOF-MS	Matrix-assisted laser desorption/ionization time-of-flight mass spectrometry
SD	Standard deviation
IQRs	Interquartile ranges
ROC	Receiver operating characteristic
AUC	Area under the curve
PPV	Positive predictive value
NPV	Negative predictive value
