# Supplementary material for: Assessment of Phoenix Sepsis Score, pSOFA, PELOD-2, and PRISM III in Pediatric Intensive Care
Source: Children (Basel). 2025 Feb 21;12(3):262. doi: 10.3390/children12030262 (PMC11941747; doi:10.3390/children12030262)
Supplement: Supplementary file 1 [file children-12-00262-s001.zip › Supplementary File S1.pdf]

Clinical Questionnaire Form for Participants in the Research Project:

"Investigation of Innovative Inflammatory Markers in Children with Septic and Critical Conditions"

Full Name: ....

Age: .... Gender: M / F

Diagnosis: ....

Date of Diagnosis: ....

Date of Inclusion in the Study: ....

Medical History:

Developmental History: Pregnancy (including complications during pregnancy)

Birth: .... Height (cm): .... Weight (kg): ....

Feeding: ....

Immunization Schedule: ....

Neuropsychological and Physical Development: ....

Past Illnesses: Is the child frequently ill? Yes / No. If Yes, how often?

....

Chronic Diseases: Does the patient have any chronic diseases?

If Yes, specify:

....

Family History: Mother – diseases: ....

Father – diseases: ....

Siblings – .... years,

Diseases:
....

Medication Use: Yes / No. If Yes, specify which and since when:

....

Allergies: Yes / No. If Yes, specify:
....

Current Complaints: Has the child had a high or low temperature in the last 7 days? Yes/No.

Does the child have a cough? Yes/No. If Yes, since when?...

Has the child had changes in breathing frequency in the last 7 days? Yes/No. If Yes, since when?
....

Has the child experienced changes or loss of consciousness/drowsiness/lethargy/seizures in the last 7 days? If Yes, since when?
....

Does the child report vision problems? Yes/No. If Yes, since when?
....

Has the child had changes in skin color/blueness of the lips/nail beds? Yes/No. If Yes, since when? ....

Has the child experienced nausea/vomiting in the last 7 days? If Yes, since when and how many times?
....

Has there been a change in the frequency and color of the child’s urine? Yes/No. If Yes, since when and specify the color:
....

Has the child been consuming enough fluids and food in the last 7 days? Yes/No.

If No, since when?
....

Prior hospitalization in the last 7 days? – Yes/No.

General Condition:

Height (cm): ………………. Weight (kg): .... BMI (kg/m^2): ………

Ponderal Index (PI kg/m^3), PI = Weight (kg)/Height^3 (m^3)..

Head – configuration: ...., Head Circumference (cm): ……………

Anterior Fontanelle: ...., Posterior Fontanelle: ....

Temperature (°C): ....

Neck – freely movable or not:
....

Thyroid Gland:
....

Skin:
....

Respiratory System – Respiratory Rate: ..../min,

Breathing: ....

....

SpO_2: ………. % at FiO_2: ....

Cardiovascular System – Heart Rate: …………………./min,

Blood Pressure: …………………..... mmHg on the left arm, pathological murmurs:
....

Abdomen: ....
Liver: ....
Spleen: ....

Nervous System – Pediatric Glasgow Coma Scale (pGCS): .... points,

Kernig's Sign: ...., Brudzinski's Sign: ....

Neonatal Reflexes:
....
....
....

Musculoskeletal System:
....
....
…………………………………………………………………………………………
Throat:
....

Nose:
....

Innovative Inflammatory Markers	
Marker	*Result*
Presepsin (pg/mL)	
sMR (ng/mL)	

Microbiological Blood Testing	
Blood Culture	*Result*
At Admission	
24 Hours Post Admission	

Blood Gas Analysis	
Parameter (Reference Values)	*Result At Admission*
pH (7.36-7.44)	
pCO_2 mmHg (35-45)	
tCO_2 mmol/L (23-29)	
tCO_2 mmol/L (23-29)	
PaO_2 mmHg (80-100)	
Lactate (mmol/L)	

Laboratory Biochemical Parameters	
Parameter (Reference Values)	*Result At Admission*
Procalcitonin (0.00-0.05) ng/mL	
Glucose (3.3-5.6) mmol/L	
Urea (3.2-8.2) mmol/L	
Creatinine (18-62) µmol/L	
Uric Acid (220-547) µmol/L	
AST (0.0-34.0) IU/L	
ALT (10.0-49.0) IU/L	
Total Bilirubin (5.0-21.0) µmol/L	
Direct Bilirubin (0.0-3.0) µmol/L	
Albumin (32.0-48.0) g/L	
CRP (0-5.0) mg/L	
Na (132-146) mmol/L	
K (3.5-6.0) mmol/L	
Cl (99-109) mmol/L	
Ca (2.18-2.60) mmol/L	

Hematological Analysis	
Parameter (Reference Values)	*Result At Admission*
Leukocytes 10^9/L	
Neutrophils %	
Lymphocytes %	
Hemoglobin (Hb) g/L	
Hematocrit (Hct) L/L	

Platelets (Plt) 10^9/L	

Coagulation	
Parameter (Reference Values)	*Result At Admission*
Prothrombin Time (PT) (11.5-14.8) sec	
aPTT (25.4-36.9) sec	
D-dimer (<0.232) mg/L	
Fibrinogen (2.38-4.98) mmol/L	
INR (0.8-1.2)	

Scoring Systems	
Score (Points)	*Result*
pSOFA	
PRISM III	
PELOD-2	
Phoenix Sepsis Score	
