# Supplementary figures and images for: Assessment of Phoenix Sepsis Score, pSOFA, PELOD-2, and PRISM III in Pediatric Intensive Care
Source: Children (Basel). 2025 Feb 21;12(3):262. doi: 10.3390/children12030262 (PMC11941747; doi:10.3390/children12030262)

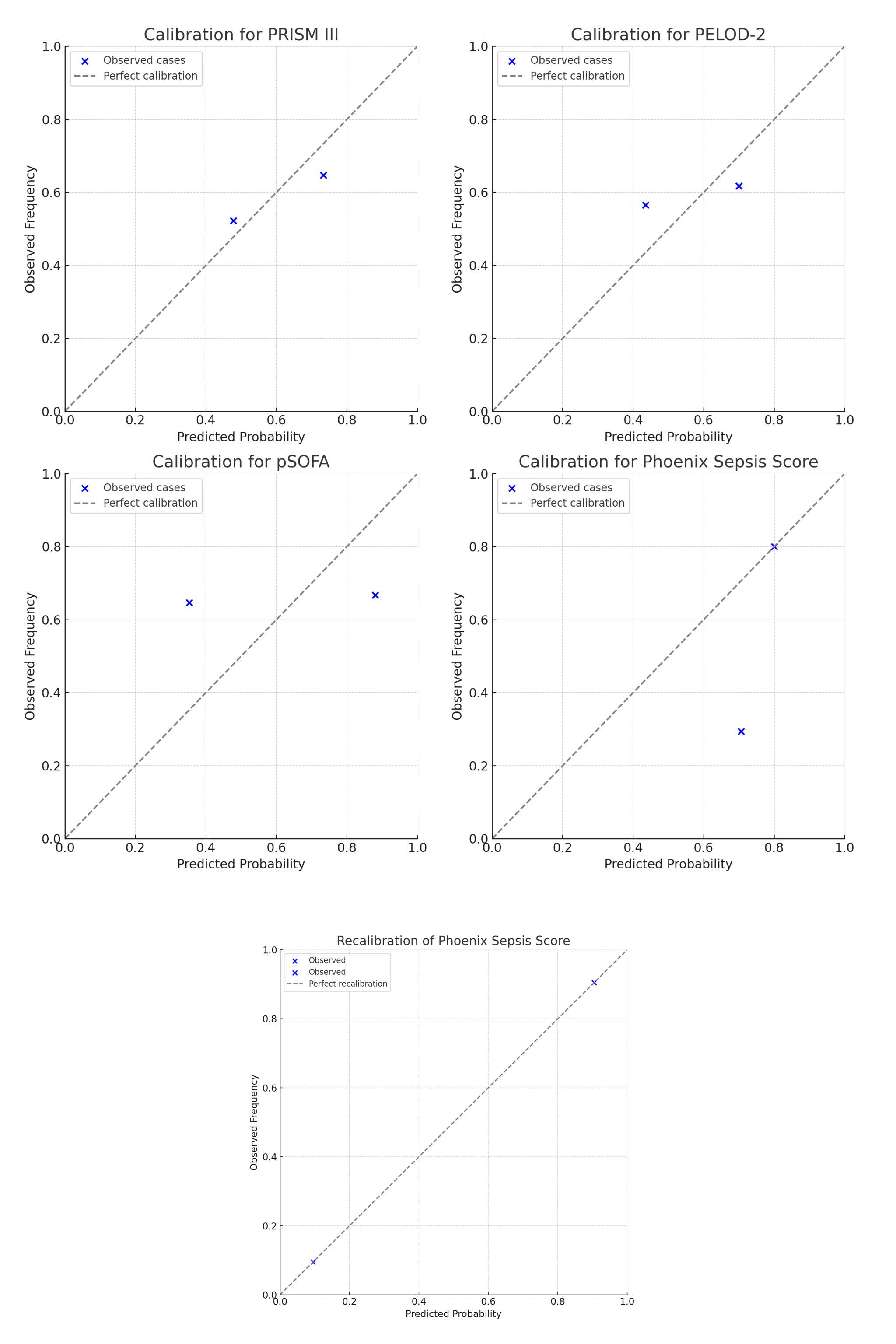

Supplement: Supplementary file 1 [file children-12-00262-s001.zip › Supplementary File S2.jpg]
